# Effect of Sex Steroids and PGF_2α_ on the Expression of Their Receptors and Decorin in Bovine Caruncular Epithelial Cells in Early–Mid Pregnancy

**DOI:** 10.3390/molecules27217420

**Published:** 2022-11-01

**Authors:** Monika Jamioł, Magdalena Sozoniuk, Jacek Wawrzykowski, Marta Kankofer

**Affiliations:** 1 Department of Biochemistry, Faculty of Veterinary Medicine, University of Life Sciences in Lublin, Akademicka Street 12, 20-033 Lublin, Poland; 2 Institute of Plant Genetics, Breeding and Biotechnology, Faculty of Agrobioengineering, University of Life Sciences in Lublin, Akademicka Street 15, 20-950 Lublin, Poland

**Keywords:** bovine pregnancy, caruncular epithelial cells, PGF_2α_ receptor, steroid hormone receptors, decorin

## Abstract

Changes in the expression of various genes, including pregnancy-associated hormone receptors and extracellular matrix proteins, have been suggested to play a significant role in bovine placental development. This study aimed to examine the influence of sex steroids and PGF_2α_ on decorin (DCN) expression in the epithelial cells of bovine caruncle in early–mid pregnancy in cows. The expression patterns of DCN, PTGFR_,_ PGR and ESR1 were analyzed by RT-qPCR and Western blotting in primary caruncular epithelial cell cultures (PCECC) and placental tissue homogenates derived from the 2nd and 4th months of pregnancy. PCECC were found to express DCN, PTGFR_,_ PGR and ESR1. The intensity of PGR staining was higher in cells derived from the 4th month of pregnancy (*p* < 0.05). The 17β-estradiol, progesterone and PGF_2α_ have not been shown to affect DCN expression. PGF_2α_ decreased PTGFR expression in cells derived from the 4th month of gestation (*p* < 0.05). In conclusion, the results of the present preliminary study showed that the expression of the PTGFR, ESR1, PGR and DCN in PCECC does not vary throughout early–mid pregnancy. Further studies should be carried out to observe the relationship between hormonal status and cellular adhesion to determine their importance for properly developing placentation and pregnancy in cows.

## 1. Introduction

Hormonal interplay and genomic regulation of expression of selected protein molecules, including adhesive proteins, are crucial for an appropriate course of pregnancy in cows. Successful pregnancy relies on appropriate cell adhesion modulated by adhesive proteins during implantation and placentation [1].

Estrogens, mainly estrone (E1) and its sulfate salt (E1S), are among the major steroid hormones produced by the placenta. In comparison to 17β-estradiol produced by the ovaries, the activity of placental E1 is visibly lower. As too-high estrogen levels are unfavorable during pregnancy, unlike progesterone, E1 may be the predominant placental estrogen with a limited estrogenic spectrum that only covers pregnancy needs. Generally, estrogens are responsible for placental growth, differentiation and functioning. Placental estrogens can act locally, since the localisation of their receptors ER_α_ has been recognized in the bovine placenta [2,3]. During the periparturient period in cows, estrogen receptors were observed in endometrial stromal cells, myometrial smooth muscle cells, endometrial surface epithelial cells and glandular epithelial cells [4].

Prostaglandin F_2α_ (PGF_2α_) has important functions in reproductive events such as ovulation, luteolysis, embryogenesis and implantation, as well as cytokine regulation and cell proliferation [5,6]. In cattle, PGF_2α_ concentrations increase in the endometrium in the first month of gestation [7], confirming its participation in the development of pregnancy. The mechanism of action of prostaglandins is different from that of steroid sex hormones. PGF_2α_ acts through the G-protein-coupled cell membrane receptor PTGFR [8]. It is known that estradiol (E_2_) stimulates the synthesis of PTGFR and PGF_2α_ synthase in the endometrium and oviduct of ruminants. PGF_2α_ synthesis is indirectly stimulated by the estrogens that upregulate COX-2 expression [9]. However, the specific role of PGF_2α_ and its mechanism of action in developing the placenta remains unclear.

Progesterone (P_4_) is the essential hormone for the maintenance of bovine pregnancy, since it controls endometrial differentiation, myometrial quiescence, and closure of the cervix, as well as the lack of immunological response [10]. Immunohistochemical localization of progesterone receptors in bovine placenta throughout mid and late gestation has already been described. From day 150 to day 270 of pregnancy, PGR-positive staining was found in the nuclei of caruncular stromal cells and caruncular vascular pericytes of the placentome [2]. A strong immunoreaction for progesterone was noticed in the nuclei of endometrial stromal cells and myometrial smooth muscle cells in the intercaruncular uterine wall of periparturient cows [4].

Appropriate transfer of cell signals requires the presence of receptors which, after binding of ligands, can express their biochemical action [11]. Therefore, the determination of the presence of receptors is crucial for starting a biochemical cascade of reactions leading to the biological answer of hormones to stimuli. Moreover, the hormonal profile during pregnancy reflects cell sensitivity and regulation of biochemical metabolism related to particular stages of pregnancy [12].

Decorin (DCN) plays a significant role during pregnancy, since it is responsible for the structural and functional integrity of the placenta and foetal membranes [13]. Synthesis of DCN, as a protein molecule involved in cell adhesion, is regulated by the genomic action of sex steroids [14,15]. Decorin occurs in the placenta of cows [16,17,18,19], but it is not known what mechanism is responsible for its expression in epithelial cells, which form the interface between the foetal and maternal parts.

Several studies have been carried out on the role of DCN and pregnancy-associated hormones in pregnant cows; however, they were focused mainly on the immunohistochemical and gene expression analyses [2,19,20,21]. Data on the presence of DCN, PGF_2α_, estrogen and progesterone receptors in epithelial primary cell cultures obtained from the maternal part of the bovine placenta during early–mid pregnancy in cows is missing.

The aim of the study was to assess the potential relationship between the expression of prostaglandin F_2α_, progesterone and estradiol receptors at the mRNA and protein levels, as well as to examine the influence of sex steroids and PGF_2α_ on DCN expression in the epithelial cells of bovine caruncle in early–mid pregnancy. 

## 2. Results

**Figure 1:**
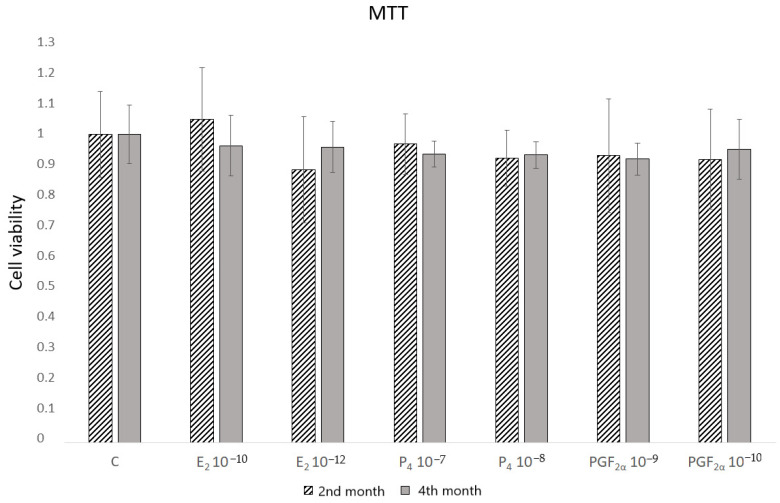
Effect of various doses of 17β-estradiol (E_2_), progesterone (P_4_) and prostaglandin F_2α_ (PGF_2α_) on caruncular epithelial cell viability in the placenta of pregnant cows measured by a MTT assay. C—vehicle. Data from two independent experiments performed in quadruplicate.

To select the doses of hormones that do not affect the cell viability, the MTT assay was conducted (Figure 1). None of the concentrations used influenced the activity of mitochondrial energy changes. In the subsequent experiment, the lowest concentrations were selected.

The transcript levels of *DCN*, *ESR1*, *PGR* and *PTGFR* were determined by RT-qPCR analysis. Amplification efficiency for analysed targets ranged from 101.30% (*PGR*) to 106.16% (*PTGFR*). The only exception was *DCN*, for which a standard curve could not be generated and analysed due to its very low transcript level in the analysed material. Consequently, in subsequent relative quantification, the *DCN* amplification efficiency was assumed to be 100%. Regression coefficients for standard curves were all above 0.99, whereas slope values oscillated around −3.2 (Supplementary Table S1).

To determine the specificity of the amplification of designed primer pairs, dissociation curves were analysed. The lack of primer–dimers formation and/or unspecific product presence was confirmed by obtaining single peaks on melting curves (Supplementary Figure S1).

Details of the expression of target genes in caruncular epithelial cells exposed to E2, P4, PGF_2α_ or PBS (control) during the 2nd and 4th months of bovine pregnancy were presented as values standing for ΔCq (Supplementary Table S2).

**Figure 2:**
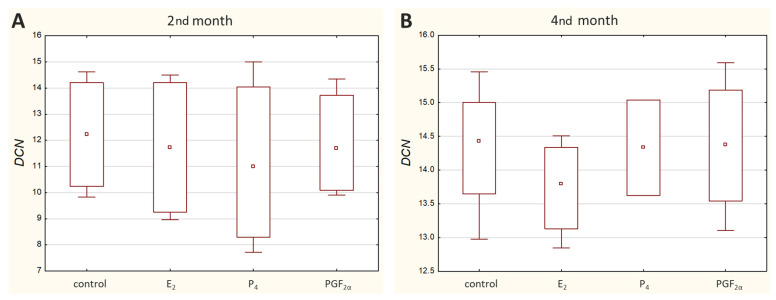
Expression of *DCN* in caruncular epithelial cells exposed to E_2_, P_4_, PGF_2α_ or PBS (control) during the 2nd (**A**) and 4th (**B**) months of bovine pregnancy. Presented values stand for ΔCq. The higher the ΔCq, the lower the mRNA level of the target gene. Data are shown in the box plots, including the minimum and the maximum value (whiskers), the sample median (small square in the middle), and the first and third quartiles (frame).

*DCN* transcripts were detected in all analysed cell cultures except for the M1 cell culture treated with P_4_, where no amplification was observed. Overall, *DCN* was noticed to be expressed at a relatively low transcriptional level, with the sample Ct ranging from 29.61 to 36.77 (Figure 2). 

No significant differences in its expression were observed under tested conditions. Its transcription upon E_2_, P_4_ or PGF_2α_ stimulation remained unchanged in comparison to the control samples (Supplementary Figure S2).

**Figure 3:**
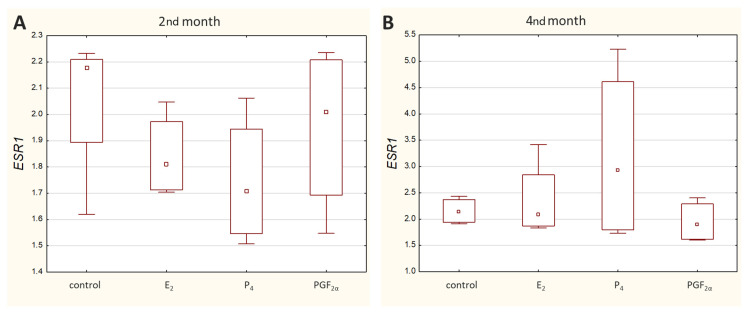
Expression of *ESR1* in caruncular epithelial cells exposed to E_2_, P_4_, PGF_2α_ or PBS (control) during the 2nd (**A**) and 4th (**B**) months of bovine pregnancy. Presented values stand for ΔCq. The higher the ΔCq, the lower the mRNA level of the target gene. Data are shown in the box plots, including the minimum and the maximum value (whiskers), the sample median (small square in the middle), and the first and third quartiles (frame).

**Figure 4:**
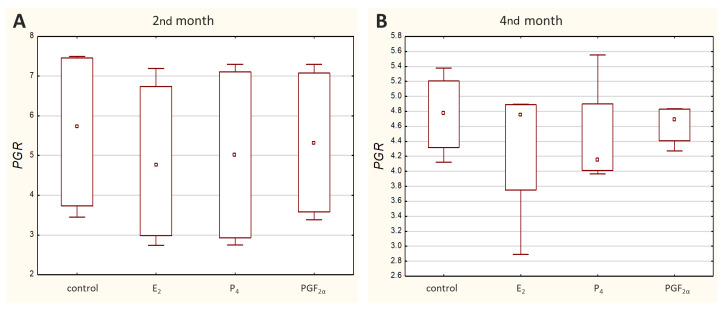
Expression of *PGR* in caruncular epithelial cells exposed to E_2_, P_4_, PGF_2α_ or PBS (control) during the 2nd (**A**) and 4th (**B**) months of bovine pregnancy. Presented values stand for ΔCq. The higher the ΔCq, the lower the mRNA level of the target gene. Data are shown in the box plots, including the minimum and the maximum value (whiskers), the sample median (small square in the middle), and the first and third quartiles (frame).

Transcripts of all other genes-of-interest (GOIs)-encoding receptors were detected in all analysed samples. Both *ESR1* (Figure 3) and *PGR* (Figure 4) displayed a similar range of sample Ct values (21.02–29.06 and 22.57–29.98, respectively). 

**Figure 5:**
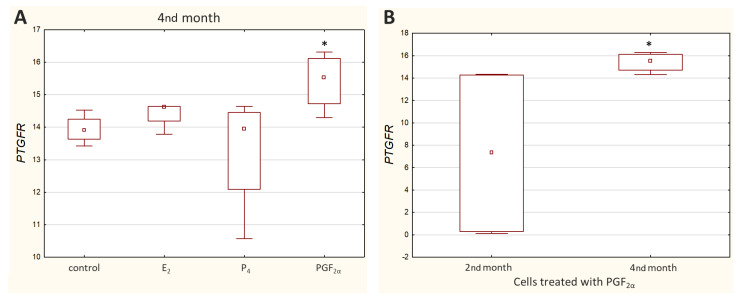
Expression of *PTGFR* in caruncular epithelial cells exposed to E_2_, P_4_, PGF_2α_ or PBS (control) during the 4th month of bovine pregnancy (**A**). Comparison of *PTGFR* expression in cells from the 2nd and 4th months of pregnancy (**B**). Presented values stand for ΔCq. The higher the ΔCq, the lower the mRNA level of the target gene. Data are shown in the box plots, including the minimum and the maximum value (whiskers), the sample median (small square in the middle), and the first and third quartiles (frame). Values with asterisks are significantly different (*p* < 0.05).

Ct values reported for *PTGFR* varied greatly depending on the cell culture (Ct value ranged from 19.57 to 35.50), with noticeably higher transcript levels observed in the M5 cell culture. Relative quantification showed a significant decrease (*p* < 0.05) in *PTGFR* expression in the cells incubated with PGF_2α_ derived from the 4th month of pregnancy (Figure 5A). 

Furthermore, a significantly higher (*p* < 0.05) transcript level of *PTGFR* was detected in the 2nd month than in the 4th month of gestation (Figure 5B) when the cells were treated with PGF_2α_. Other results that were not statistically significant are presented in supplementary Figure S2.

**Figure 6:**
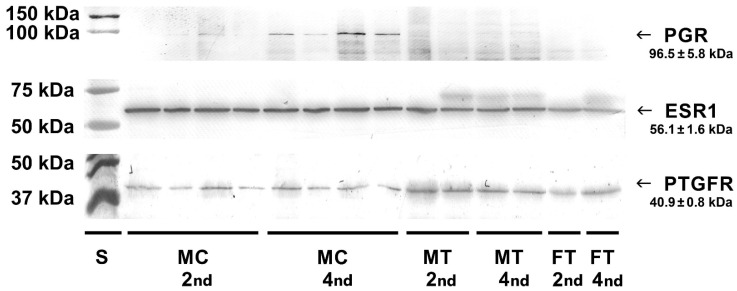
The presence of progesterone, estrogen and prostaglandin F_2α_ receptors in bovine caruncular epithelial cells and placental tissues at early gestational stages was demonstrated by Western blotting. PGR—progesterone receptor; ESR1—estrogen receptor; PTGFR—prostaglandin F_2α_ receptor; MC—caruncular epithelial cells; MT—placental tissue from the maternal part of the placenta; FT—placental tissue from the foetal part of the placenta.

Western blot analysis of cell lysates and homogenates of bovine placentomes was carried out to detect protein receptors for selected hormones. Specific bands for the progesterone receptor (96.5 kDa), estrogen receptor (56.1 kDa) and prostaglandin F_2α_ receptor (40.9 kDa) were detected in examined cell lysates and tissue homogenates (Figure 6).

The original Western blot image is presented in Supplementary Figure S3.

## 3. Discussion

Studies on the effects of various factors on maternal–foetal connections in bovine placenta require the use of in vitro models. To confirm that cell cultures respond to the action of signalling molecules, it is necessary to verify that the cells express receptors for them.

The present manuscript confirmed not only the presence of receptors of sex steroids and PGF_2α_ but also decorin—which is novel—in early–mid pregnant bovine epithelial cells. PTGFR, PR, ER_α_ and decorin mRNAs were expressed in caruncular epithelial cells derived from the 2nd and 4th months of pregnancy. These results suggest that in pregnant cattle, caruncular epithelial cells in early–mid pregnancy are under the control of progesterone, estradiol and prostaglandin F_2α._

The binding of hormones to the receptor is a crucial reaction for the expression of the biological answer of hormones [11]. Receptors are of protein character, and their synthesis is genetically determined, while their regulation is hormonally controlled [22]. In some cases, mRNA presence does not mean protein synthesis [23]. To be sure that gene expression is complete and the presence of mRNA guarantees protein synthesis, the determinations of both products—mRNA and protein—are necessary [24]. This study confirmed that mRNA expression was utilised for appropriate protein synthesis as it was visible in Western blotting both in cell lysates and tissue homogenates.

Hormonal profile during pregnancy reflects different stages of foetal and placental development, as well as appropriate reactions of cells. The culture of cells with the presence of selected hormones may help establish the mechanisms of hormonal actions.

In early pregnancy, PGF_2α_ concentrations increase significantly in the endometrium [6]. Ulbrich et al. (2009) showed that between days 12–15 of gestation, PGF_2α_ concentration in the bovine uterus of cows increases more than 30-fold [25]. As pregnancy progresses, PGF_2α_ levels also increase in foetal fluids. Between 60 and 111 days of gestation, PGF_2α_ levels in the uterine and amniotic fluids were 9.0–11.7 ng/mL and 2.4–5.7 ng/mL, respectively [26].

The literature lacks detailed data on the PGF_2α_ concentration in the plasma of pregnant cows due to the short half-life of the molecule. Moreover, studies on prostaglandin F_2α_ receptor patterns in the bovine placenta are also scarce. In this study, no effect of PGF_2α_ on the expression of its receptor was observed in the 2nd month, which may suggest a decreased sensitivity of cells to this hormone in the earlier stage of pregnancy. In turn, decreased expression of *PTGFR* under the influence of PGF_2α_ in the 4th month of pregnancy may be the result of a cell feedback response. A similar dependence occurs in the case of the action of progesterone. The continuous exposure of the uterine endometrium to progesterone regulates the expression of these receptors in the epithelium, leading to a reduction in their synthesis in early pregnancy in many mammals, including cows [27]. On the other hand, some studies have shown that there is a positive correlation between bovine PGF_2α_ concentration and the expression of its receptor during the oestrous phase [28,29]. In this study, *PTGFR* mRNA expression in non-treated cells did not differ significantly between the 2nd and 4th months of pregnancy in caruncular epithelial cells. Similar results obtained at other stages of pregnancy have been observed in the study of Sakumoto et al. (2014). There was no difference in *PTGFR* expression between the corpus luteum of non-pregnant cows and animals in the 1st–2nd months of pregnancy. However, the prostaglandin F_2α_ receptor mRNA level was increased in the 5th month of pregnancy [30]. 

Nowadays, it is not fully understood how the levels of steroid hormones, including estrogens, are established in maternal blood during pregnancy in cows. Nevertheless, circulating estrogen concentrations increase during pregnancy, initially slowly after the 2nd month of pregnancy (from day 60 to 80). The first sharp increase in estrogen levels does not begin until around day 100–120. Thereafter, the concentrations continue to rise, but at a more gradual rate, up to the 8th month of gestation. From around day 250 to the end of pregnancy, estrogen levels increase sharply again, peaking around 2–5 days before delivery, and then dropping sharply at term [31]. In the bovine uterine wall around parturition, endometrial epithelial cells revealed more intense estrogen receptor immunostaining compared to endometrial stromal cells [4]. Moreover, Schuler et al. (2002) analyzed the location and distribution of ER_α_ in the placentas of cows within 150–270 days of gestation using immunohistochemistry. According to the results obtained in the caruncular epithelium, the primary antibodies recognizing the C-terminus gave positive nuclear and cytoplasmic signals [2]. Our research focused on the earlier stage of pregnancy. In this study, in caruncular epithelial cells and placental tissues, the primary antibodies recognizing the middle of the sequence gave also positive signals. No changes in staining intensity were observed between the 2nd and 4th months of pregnancy taking into account homogenates of the maternal part of the placenta, however with a tendency to be decreased in the homogenates of the foetal part. Western blotting analyses in tissue homogenates containing different cell types were confirmed by the determinations in epithelial cell lysates stating that these cells exhibit the presence of protein for examined receptors. 

Peripheral blood progesterone levels in cows remain relatively constant during pregnancy—in the range of 5–10 ng/mL. In early pregnancy, there is an intense increase in blood progesterone levels. A slight decrease is observed in mid-pregnancy (from day 100 to 180). Around day 200 of pregnancy, progesterone levels rise again, but only slightly, and then they begin to decrease gradually in the 60 days prior to parturition, with a sharp decrease from around day 2 before calving [31,32,33]. In the present study, the antibodies that detect the B form of the progesterone receptor were used. Interestingly, there were no differences in *PGR* transcript levels in cultured cells between examined time periods, while its protein showed a visually more intense signal in the 4th month of gestation compared to the 2nd month. Moreover, homogenates of placental tissues did not exhibit positive PGR staining at all, which is in line with the results obtained by others. Immunohistochemical analysis of bovine placentomes throughout mid- and late gestation (from the 5th to the 9th month) showed no PGR-positive caruncular epithelial cells, while positive nuclear immunostaining was observed in maternal stromal cells and vascular pericytes. In placentomes from cows at term, casual positive nuclear staining was also noticed in the walls of small caruncular arteries [21]. What is more, in the bovine intercaruncular uterine wall around parturition, the endometrial progesterone receptor protein was not found in the epithelial layer while glands revealed a very weak immunoreaction [4]. The presence of progesterone receptors in epithelial cells in the 2nd and 4th months of pregnancy may suggest that this placental layer responds to progesterone in the early months of pregnancy rather than in the later period.

The presence of decorin (DCN) protein in bovine caruncular epithelial cells in the 2nd and 4th months of pregnancy was previously confirmed by our team [16]. Since the expression of extracellular matrix (ECM) proteins is influenced by progesterone [34,35], we took into consideration the possible impact of selected hormones related to pregnancy on *DCN* mRNA expression. Our present studies did not reveal any effect of selected hormones on the expression of decorin in the examined period of time. However, it has to be taken into account that the expression of decorin mRNA was at a very low level, which makes it difficult to draw clear conclusions. We hypothesized that the influence of PGF_2α_ and P_4_ on the anti-adhesive properties of decorin [18] may be related to their influence on decorin expression. Unfortunately, based on the results obtained here, this mechanism cannot be confirmed. The low *DCN* mRNA expression presented here is in agreement with previously reported findings in cows. According to Guillomot et al. (2014), its expression tended to increase in the endometrial stroma only in the 1st month of gestation and decreased in the 2nd month. Interestingly, the authors did not detect DCN protein in the placentome layers that expressed decorin mRNA [19]. On the other hand, our previous research results showed clearly intense protein bands during the WB analysis [16], although the mRNA level examined here was low. Moreover, in the study of Franczyk et al. (2018), the ELISA test confirmed quantitatively the presence of DCN protein in tissue homogenates in early–mid bovine placenta. Indeed, the test showed that the concentration of decorin during pregnancy was significantly lower in the maternal part of the placenta (67.54 pg/mg protein) compared with the foetal part (177.54 pg/mg protein) [17]. All of these together demonstrate the complexity of controlling the synthesis and degradation of not only protein but also mRNA. The amount of each mRNA to be expressed is actually controlled and determined by the cell, which depends on the cell status at any given time. The exact mechanisms underlying ECM metabolism, including the network of signalling pathways, need to be clarified.

In conclusion, the results of the present preliminary study showed that the expression of *PTGFR*, *ESR1*, *PGR* and *DCN* in the bovine caruncular epithelial cells does not vary over the course of early–mid pregnancy. Further studies should be carried out to observe the relationship between hormonal status and cellular adhesion to determine their importance for properly developing placentation and pregnancy in cows.

## 4. Materials and Methods

### 4.1. Cell Cultures

Four primary cell cultures originating from bovine caruncles (two from the 2nd month of pregnancy, and two from the 4th month of pregnancy) were used to examine target genes expression. These were the same cells used in a parallel experiment described in detail earlier by Sozoniuk et al. (2022) [36]. The cells were cultured in a medium containing DMEM/F-12 50:50 (15-090-CVR, Corning, Stryków, Poland), 10% FBS (35-079-CV, Corning), 100 IU/mL penicillin/100 μg/mL streptomycin (30-002-CI, Corning) and 2 mM L-glutamine (G7513, Sigma-Aldrich, Poznań, Poland) as described previously [16,18]. 

### 4.2. Tissue Homogenates

Placental tissues were collected in a local slaughterhouse from healthy, pregnant HF cows (2nd and 4th month of pregnancy, n = 8). The age of the animals ranged from 4 to 6 years. Pregnancy age was assessed based on the crown-rump-length (CRL) of the foetus. Placental tissue samples were collected from a similar location in the uterus and within the placentome. Placentomes (1 g) were manually separated into maternal and foetal parts with great care to avoid any contamination by the foetal part and homogenized (4 °C, 20 min, 6500× *g*) in 2 mL 0.05 M phosphate buffer (pH = 7.2) containing Triton X-100 and in the presence of protease inhibitor cocktail (87785, Thermo Scientific™, Warszawa, Poland) using Ultra-Turrax T 25 (Ikawerk, Janke and Kunkel Inc., Staufen, Germany). Supernatants were frozen (−20 °C) for further analysis. Maternal and foetal parts of the placenta were analysed separately.

### 4.3. Mitochondrial Oxidative Activity Test

Cell viability based on measuring the activity of energy changes in the mitochondria was assessed using the MTT assay according to the method of Mosmann (1983) with some modifications [37]. The cells, at a density of 0.5 × 10^6^ cells/mL, were seeded in 96-well plates and cultured (37 °C, 5% CO_2_) in a medium for 24 h. The 50,000 cells suspended in 100 µL medium were seeded per well. The next day, the culture medium was changed to the medium without FBS and the cells were incubated (37 °C, 5% CO_2_) for the next 24 h. Thereafter, the medium was replaced with 100 µL medium containing E_2_ (E2758, Sigma-Aldrich) at concentrations: 10^−12^ mol/L and 10^−10^ mol/L; P_4_ (Prolutex^®^, IBSA, Lublin, Poland) at concentrations: 10^−7^ mol/L and 10^−8^ mol/L; PGF_2α_ (Enzaprost F) at concentrations: 10^−9^ mol/L and 10^−10^ mol/L; or vehicle (PBS), and incubated (37 °C, 5% CO_2_) for 24 h. The doses were physiological and selected based on the research of others [31,38,39,40,41]. The 20 µL MTT (M5655, Sigma-Aldrich) at a concentration of 5 mg/mL (in PBS) was added to 100 µL medium and the plate was incubated (37 °C, 5% CO_2_) for 2 h. After washing with PBS, the formazan crystals were precisely dissolved with 100 µL DMSO (D8418, Sigma-Aldrich) by the use of a rotating mixer. The absorbance was measured at 550 nm using a microplate reader (Labsystems Multiskan RC, Warszawa, Poland). The results were calculated as % of control, and cell viability was expressed in absolute values (control = 1). Two independent experiments were performed in quadruplicate.

### 4.4. Preparation of Cell Lysates for Transcriptomic Analysis

The cells, at a density of 0.5 × 10^6^ cells/mL, were seeded in 6-well plates and cultured (37 °C, 5% CO_2_) in a medium for 24 h. The next day, the culture medium was changed to the medium without FBS and the cells were incubated (37 °C, 5% CO_2_) for the next 24 h. Thereafter, the cells were exposed to 10^−12^ mol/L E_2_, 10^−8^ mol/L P_4_, 10^−10^ mol/L PGF_2α_ or vehicle (PBS). For this purpose, the medium was replaced with 2 mL fresh medium containing the abovementioned hormones or buffer and incubated (37 °C, 5% CO_2_) for 24 h before lysis. After discarding the medium, the wells were washed with 5 mM EDTA/PBS and the cells were then trypsinized (0.5% trypsin in 5 mM EDTA/PBS) until detached. The cells were then carefully resuspended in a culture medium and transferred to the centrifuge tubes. After centrifugation (200× *g*, 5 min, RT), cell pellets were washed with PBS and centrifuged again under the same conditions. Supernatants were discarded and 0.5 mL lysis buffer (pH = 7.5; 100 mM Tris, 500 mM LiCl, 10 mM EDTA, 1% SDS, 5 mM DTT) with 0.5 μL RNase inhibitor (EO0381, Thermo Scientific™) was added to the cell pellets, followed by pipetting up and down a couple of times to ensure complete lysis. The resulting foam was reduced by short 30 s centrifugation. The lysates were frozen and stored at −80 °C for later isolation of mRNA.

### 4.5. Preparation of Cell Lysates for Proteomic Analysis

The cells, at a density of 0.25 × 10^6^ cells/mL, were seeded in 75 cm^2^ flasks and incubated (37 °C, 5% CO_2_) in a medium until 80% confluence was reached (approx. 5 days). The culture medium was discarded and cells were washed 3× with 10 mL PBS with 2 μL protease inhibitor cocktail (87785, Thermo Scientific™). Afterwards, 1 mL lysis buffer (GA-701-001, GeneAll, Lublin, Poland) with 2 µL protease inhibitor cocktail was thoroughly distributed on the bottom of the flask and kept on ice for 5 min. The cells were then harvested using a cell scraper (353089, Falcon, Warszawa, Poland), centrifuged (16,000× *g*, 10 min, 4 °C), and obtained supernatants were frozen and stored at −20 °C for later electrophoretic separation.

Protein concentration in the cell lysates was measured using an Infrared Spectrometer (Direct Detect^®^, Merck, Poznań, Poland) and polytetrafluoroethylene membrane cards (DDAC00010, Merck) according to the manufacturer’s instructions.

### 4.6. Isolation of mRNA from the Cell Lysate

mRNA isolation was performed using Dynabeads^®^ Oligo (dT)_25_ (61005, Invitrogen™, Warszawa, Poland) according to the manufacturer’s instructions with minor modifications. The beads were thoroughly resuspended in the vial using a vortex to obtain a uniform brown suspension. A total of 500 μL of the beads were transferred to a 1.5 mL tube and placed on a magnet (Merck Millipore PureProteome™ Magnetic Stand) for 2 min. After discarding the supernatant, the tube was removed from the magnet and 500 μL binding buffer (pH = 7.5; 20 mM Tris, 1 M LiCl, 2 mM EDTA) was added to suspend the beads. The tube was placed on the magnet for 2 min. After discarding the supernatant, the tube was removed from the magnet and 500 μL binding buffer was added again. The tube was placed on the magnet for the next 2 min. The solution was removed from the washed beads and then 0.5 μL lysate was added and mixed with the beads. mRNA annealing to the oligo dT sequence was performed by rotating on a mixer for 8 min at RT. The vial was placed on the magnet for 2 min and the supernatant was removed. The beads were then thoroughly washed at RT using a magnet to separate the beads with mRNA from the supernatant, 1 × 1 mL with washing buffer A (pH = 7.5; 10 mM Tris, 0.15 M LiCl, 1 mM EDTA, 0.1% SDS) and then 1 × 1 mL and 1 × 0.5 mL with washing buffer B (pH = 7.5; 10 mM Tris, 0.15 M LiCl, 1 mM EDTA). Afterwards, 20 μL 10 mM Tris-HCl (pH = 7.5) with 0.5 μL RNase inhibitor was added to elute the mRNA from the beads. The tube was incubated for 4 min at 80 °C and then placed on the magnet, and the supernatant containing the mRNA was quickly transferred to a new tube placed in ice. The total amount of extracted mRNA was quantified by spectrophotometric determination of the optical density at 260 nm (NanoDrop 2000, Thermo Scientific^TM^). Obtained mRNA was subjected to gene expression analysis.

### 4.7. RT-qPCR

The DNA contamination removal and reverse transcription were performed with the use of Maxima First Strand cDNA Synthesis Kit for RT-qPCR, with dsDNase (K1671, Thermo Scientific™) according to the manufacturers’ instructions. The synthesis of cDNA was carried out on 0.5 µg of mRNA in a total reaction volume of 20 µL. Obtained cDNA was used as a template in qPCR reactions. Before gene expression analysis, reference gene selection was carried out on 10 potential reference genes [36]. Based on the average expression stability value (M value) and V_n_/V_n+1_ index calculated by the geNorm algorithm, two best-performing reference genes were chosen for the normalization of the data (*ACTR1A* and *HDAC1*). Quantitative PCR was performed on QuantStudio 3 apparatus (Applied Biosystems™, Warszawa, Poland) using PowerUp™ SYBR™ Green Master Mix (A25777, Applied Biosystems™). The total reaction volume of 20 µL contained either 2.5 ng or 25 ng of cDNA and 200 nM of each primer. Reaction component details and primer sequences are compiled in Table 1. The thermal profile of reactions was as follows: 2 min at 50 °C, 2 min at 95 °C, 40 cycles of 15 sec at 95 °C and 1 min at 60 °C. To confirm amplification specificity, a dissociation step was added at the end of each reaction with continuous data collection from 60 °C to 95 °C. Each reaction was carried out in two technical replications. Reverse transcriptase minus (RT-) negative control, along with No template control (NTC) was also included in the analyses. Standard curves were generated from the serial dilution of pooled cDNA. Obtained data were analysed using a dedicated relative quantification software module from ThermoFisher Cloud (ThermoFisher Scientific, Waltham, MA, USA). 

### 4.8. Western Blotting

Proteins were separated on polyacrylamide gels using the method of Laemmli (1970) [42] with some modifications. The samples were prepared as described previously [16]. The electrophoretic separation was performed for 1 h 50 min at 100 V (Mini- PROTEAN^®^ Tetra cell, Bio-Rad, Warszawa, Poland). After electrophoresis, the proteins were transferred to a PVDF membrane by the use of Criterion™ Blotter (Bio-Rad) at 100 V for 1 h 30 min, according to the method of Towbin et al. (1979) [43]. Afterwards, the membranes were blocked with CarboFree Blocking Solution (SP-5040, Vector Labs, Janki, Poland), in PBS with 0.05% Tween (PBST) for 1 h (RT). After discarding the solution, primary antibodies diluted in PBST were added and the membranes were incubated overnight (4 °C). Polyclonal rabbit estrogen receptor (1:000, LS-C167830, LSBio; at least 90% immunogen sequence identity with cow), polyclonal rabbit prostaglandin F_2α_ receptor (1:1000, LS-A1049, LSBio; at least 81% immunogen sequence identity with cow) and monoclonal mouse progesterone receptor (1:2000, GTX22765, GeneTex; anti-bovine) antibodies were used. The addition of primary antibodies was omitted in the negative control. The membranes were washed 3 × 10 min with PBST and then incubated with secondary antibodies diluted in PBST for 2 h (RT). Depending on the origin of the primary antibodies, goat Anti-Rabbit IgG (1:2000, ab6722, Abcam) or Goat Anti-Mouse IgG (1:2000, 610-1519, Rockland) conjugated with alkaline phosphatase were used. After washing in PBST (3 × 10 min), the membranes were incubated with a substrate containing nitro blue tetrazole chloride (75 mg/mL in 10% DMSO) and 5-bromo-4-chloro-3-indolyl phosphate (50 mg/mL in 10% DMSO) in substrate buffer (pH = 9.5; 0.1 M Tris, 0.1 M NaCl, 50 mM MgCl_2_) until the bands were visualised. The enzyme reaction was stopped by the washing with H_2_O. Dried membranes were scanned with GS-710 Calibrated Imaging Densitometer (Bio-Rad), and analyzed with the Quantity One 4.1 software (Bio-Rad). The molecular masses of the bands were compared with the Precision Plus Protein™ Dual Color Standard (1610374, Bio-Rad). Two independent experiments were performed in duplicate.

### 4.9. Statistical Analysis

The data was analysed using STATISTICA Version 13.0 (StatSoft, Poland, TIBCO Software Inc., Palo Alto, CA, USA). The analysis of the heterogeneity of the samples showed the lack of a normal distribution of the results; therefore, statistical non-parametric tests were selected for the analysis. Kruskal–Wallis [44] and Mann–Whitney *U* [45] tests were used to evaluate differences between groups. A probability value of <0.05 was considered to be statistically significant.

## Figures and Tables

**Table 1:** Primer sequences and qPCR reaction details.

Gene	GenBank Accession Number	Primer Sequence	Product Size (bp)	cDNA Amount per Reaction
*DCN*	NM_173906.4	F: TGCGAGTTGTCCAGTGTTCT R: AATGCCCCAGGGCTGATTTT	190	25 ng
*ESR1*	NM_001001443.1	F: TGCCTGGCTAGAGATCCTCA R: CGGAACCGAGACGAAGTAGC	165	2.5 ng
*PTGFR*	NM_181025.3	F: TTGGGCACCTCATCAATGGAA R: AAAGTGGGCACAGACCAGAG	135	25 ng
*PGR*	NM_001205356.1	F: CTACCTTAGGCCGGATTCAGA R: TGCAATCGTTTCTTCCAGCACAT	200	2.5 ng
*ACTR1A*	NM_001193248.3	F: AAGTCTGACATGGACCTGCG R: TCTTCACGTCTTTCGGAGCC	130	2.5 ng
*HDAC1*	NM_001037444.2	F: TTACGACGGGGATGTTGGAA R: GGCTTTGTGAGGGCGATAGA	136	2.5 ng

## Data Availability

Raw data are available from the first author.

## Supplementary Materials

The following supporting information can be downloaded at: https://www.mdpi.com/article/10.3390/molecules27217420/s1, Figure S1: Melting curves obtained for genes of interest and reference genes; Figure S2: Expression of *PTGFR* in caruncular epithelial cells exposed to E_2_, P_4_, PGF_2α_ or PBS (control) during the 2nd month of bovine pregnancy. Presented values stand for ΔCq. The higher the ΔCq the lower the mRNA level of the target gene. Data are shown in the box plots including the minimum and the maximum value (whiskers), the sample median (small square in the middle), and the first and third quartiles (frame); Figure S3: The original Western blot image; Table S1: Parameters derived from RT-qPCR analysis—standard curve slope, regression coefficient (R2 ), reaction efficiency, melting temperature of the amplicon (Tm) and sample Ct data; Table S2: Expression of target genes in caruncular epithelial cells exposed to E2, P4, PGF2α or PBS (control) during the 2nd and 4th month of bovine pregnancy. The presented values stand for ΔCq.
