# Supplementary material for: Effect of Sex Steroids and PGF2α on the Expression of Their Receptors and Decorin in Bovine Caruncular Epithelial Cells in Early–Mid Pregnancy
Source: Molecules. 2022 Nov 1;27(21):7420. doi: 10.3390/molecules27217420 (PMC9653824; doi:10.3390/molecules27217420)
Supplement: Supplementary file 1 [file molecules-27-07420-s001.zip › Supplementary Figure S1.pdf]

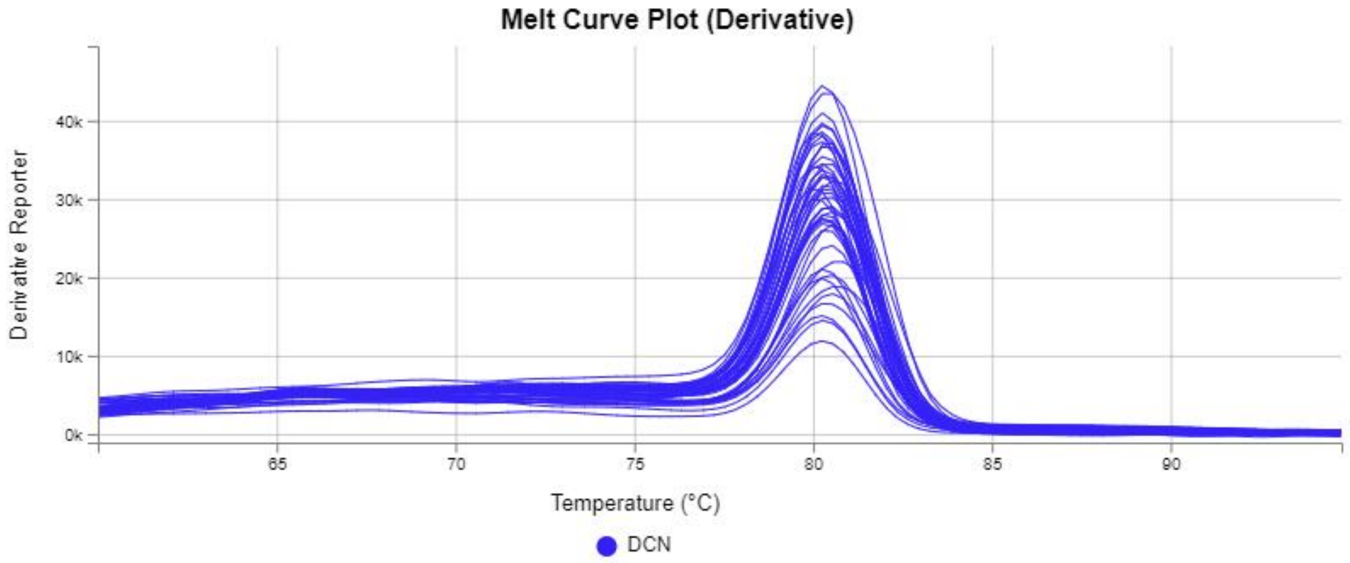
Melt Curve Plot (Derivative)
Derivative Reporter
40k
30k
20k
10k
0k
65
70
75
80
85
90
Temperature (°C)
DCN

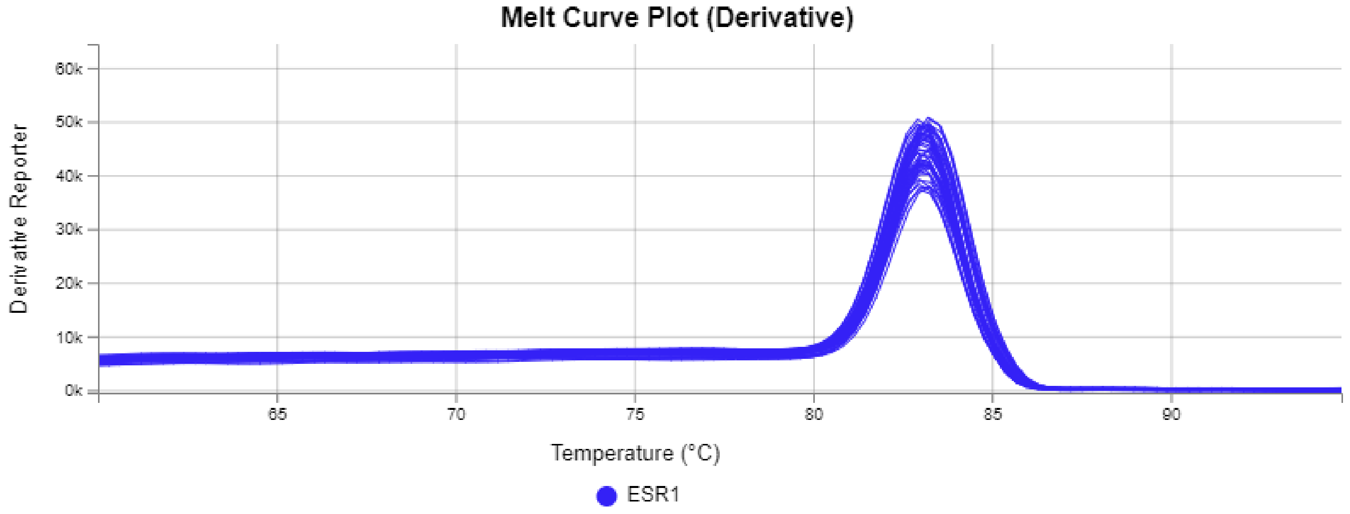
Melt Curve Plot (Derivative)
Derivative Reporter
60k
50k
40k
30k
20k
10k
0k
65
70
75
80
85
90
Temperature (°C)
ESR1

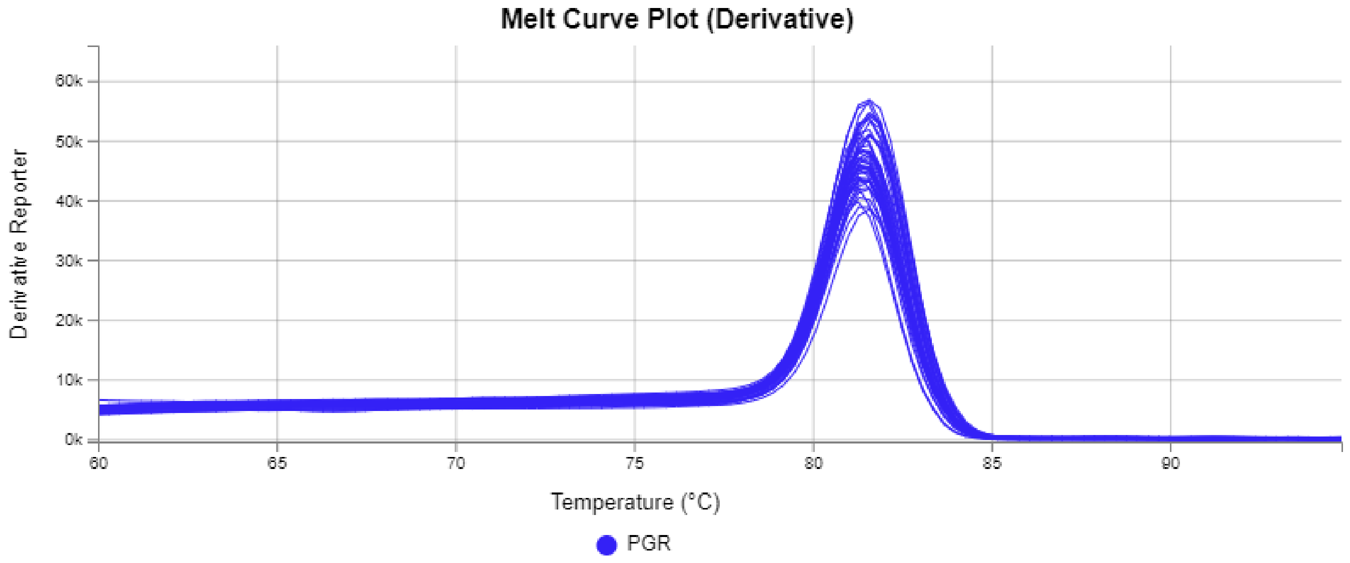
Melt Curve Plot (Derivative)
Derivative Reporter
60k
50k
40k
30k
20k
10k
0k
60
65
70
75
80
85
90
Temperature (°C)
PGR

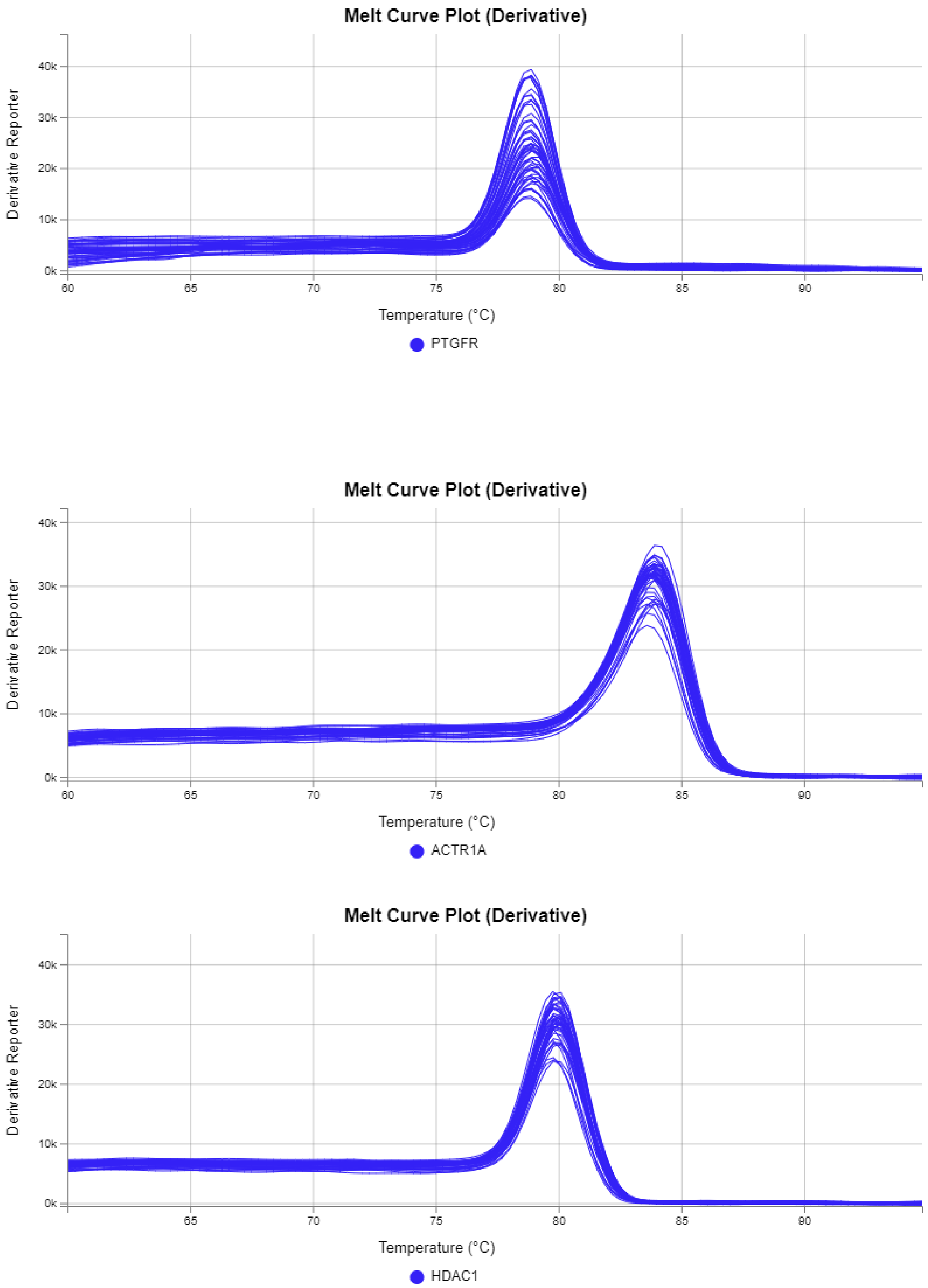

Figure S1. Melting curves obtained for genes of interest and reference genes.
