# Supplementary material for: Effect of Sex Steroids and PGF2α on the Expression of Their Receptors and Decorin in Bovine Caruncular Epithelial Cells in Early–Mid Pregnancy
Source: Molecules. 2022 Nov 1;27(21):7420. doi: 10.3390/molecules27217420 (PMC9653824; doi:10.3390/molecules27217420)
Supplement: Supplementary file 1 [file molecules-27-07420-s001.zip › Supplementary Figure S2_18.10.22.pdf]

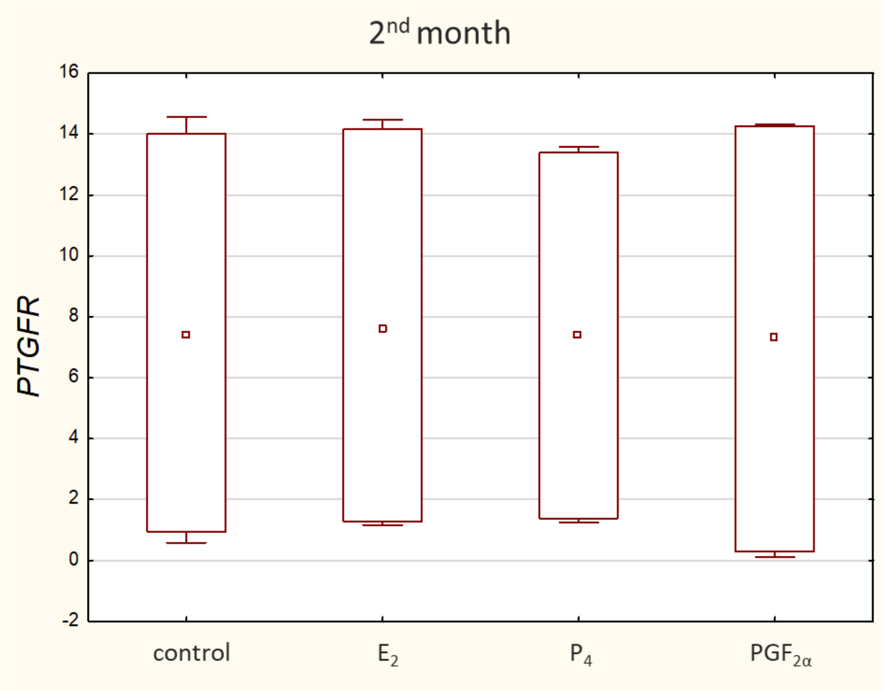

Figure S2. Expression of *PTGFR* in caruncular epithelial cells exposed to E_2, P_4, $PGF_{2\alpha}$ or PBS (control) during the 2nd month of bovine pregnancy. Presented values stand for ΔCq. The higher the ΔCq the lower the mRNA level of the target gene. Data are shown in the box plots including the minimum and the maximum value (whiskers), the sample median (small square in the middle), and the first and third quartiles (frame).
