# Supplementary material for: Effect of Sex Steroids and PGF2α on the Expression of Their Receptors and Decorin in Bovine Caruncular Epithelial Cells in Early–Mid Pregnancy
Source: Molecules. 2022 Nov 1;27(21):7420. doi: 10.3390/molecules27217420 (PMC9653824; doi:10.3390/molecules27217420)
Supplement: Supplementary file 1 [file molecules-27-07420-s001.zip › Supplementary Table S1.pdf]

Table S1. Parameters derived from RT-qPCR analysis - standard curve slope, regression coefficient (R^2), reaction efficiency, melting temperature of the amplicon (Tm) and sample Ct data.

**Target**	**Slope**	**R^2**	**Efficiency (%)**	**Tm**	**Ct_{min}**	**Ct_{max}**	**Ct range**
DCN	*NA	*NA	*NA	81°C	29.61	36.77	5.84
ESR1	-3.23	0.9985	103.87	83°C	21.02	29.06	8.04
PGR	-3.29	0.9936	101.30	81°C	22.57	29.98	7.41
PTGFR	-3.18	0.9938	106.16	79°C	19.57	35.50	15.93
ACTR1A	-3.19	0.9975	105.66	84°C	19.03	24.87	5.84
HDAC1	-3.29	0.9976	101.38	80°C	19.32	25.62	6.30

*NA - not analyzed
