# Supplementary material for: Effect of Sex Steroids and PGF2α on the Expression of Their Receptors and Decorin in Bovine Caruncular Epithelial Cells in Early–Mid Pregnancy
Source: Molecules. 2022 Nov 1;27(21):7420. doi: 10.3390/molecules27217420 (PMC9653824; doi:10.3390/molecules27217420)
Supplement: Supplementary file 1 [file molecules-27-07420-s001.zip › Supplementary Table S2.pdf]

Table S2. Expression of target genes in caruncular epithelial cells exposed to E2, P4, $PGF_{2\alpha}$ or PBS (control) during the 2nd and 4th month of bovine pregnancy. The presented values stand for ΔCq.

	2nd month				4th month			
	DCN	*ESR1*	*PGR*	*PTGFR*	*DCN*	*ESR1*	*PGR*	*PTGFR*
control	13,789	1,62	3,453	13,458	14,543	1,914	5,034	13,843
control	14,623	2,232	4,014	14,574	12,978	1,966	5,376	13,975
control	9,825	2,167	7,416	1,315	15,459	2,312	4,511	14,529
control	10,642	2,187	7,494	0,584	14,321	2,433	4,123	13,419
E2	13,934	1,704	3,226	13,863	12,847	1,831	4,609	14,592
E2	14,507	2,047	2,736	14,482	14,508	2,262	4,898	14,644
E2	9,536	1,897	6,293	1,164	14,171	3,419	4,884	13,789
E2	8,965	1,722	7,192	1,382	13,411	1,91	2,89	14,647
P4	15,012	1,586	3,119	13,591	15,04	1,866	4,247	14,641
P4	13,07	1,507	2,745	13,228	13,625	1,73	4,053	14,273
P4	7,71	2,062	6,916	1,514	undetermined	5,23	5,552	13,607
P4	8,882	1,827	7,296	1,243	undetermined	3,992	3,965	10,563
$PGF_{2\alpha}$	14,352	1,547	3,774	14,331	13,108	1,601	4,275	15,92
$PGF_{2\alpha}$	13,094	2,179	3,386	14,225	13,981	2,403	4,828	14,297
$PGF_{2\alpha}$	10,28	1,838	7,292	0,458	14,785	2,176	4,837	15,142
$PGF_{2\alpha}$	9,903	2,236	6,858	0,116	15,591	1,629	4,544	16,31
