# Supplementary figures and images for: Effect of Sex Steroids and PGF2α on the Expression of Their Receptors and Decorin in Bovine Caruncular Epithelial Cells in Early–Mid Pregnancy
Source: Molecules. 2022 Nov 1;27(21):7420. doi: 10.3390/molecules27217420 (PMC9653824; doi:10.3390/molecules27217420)

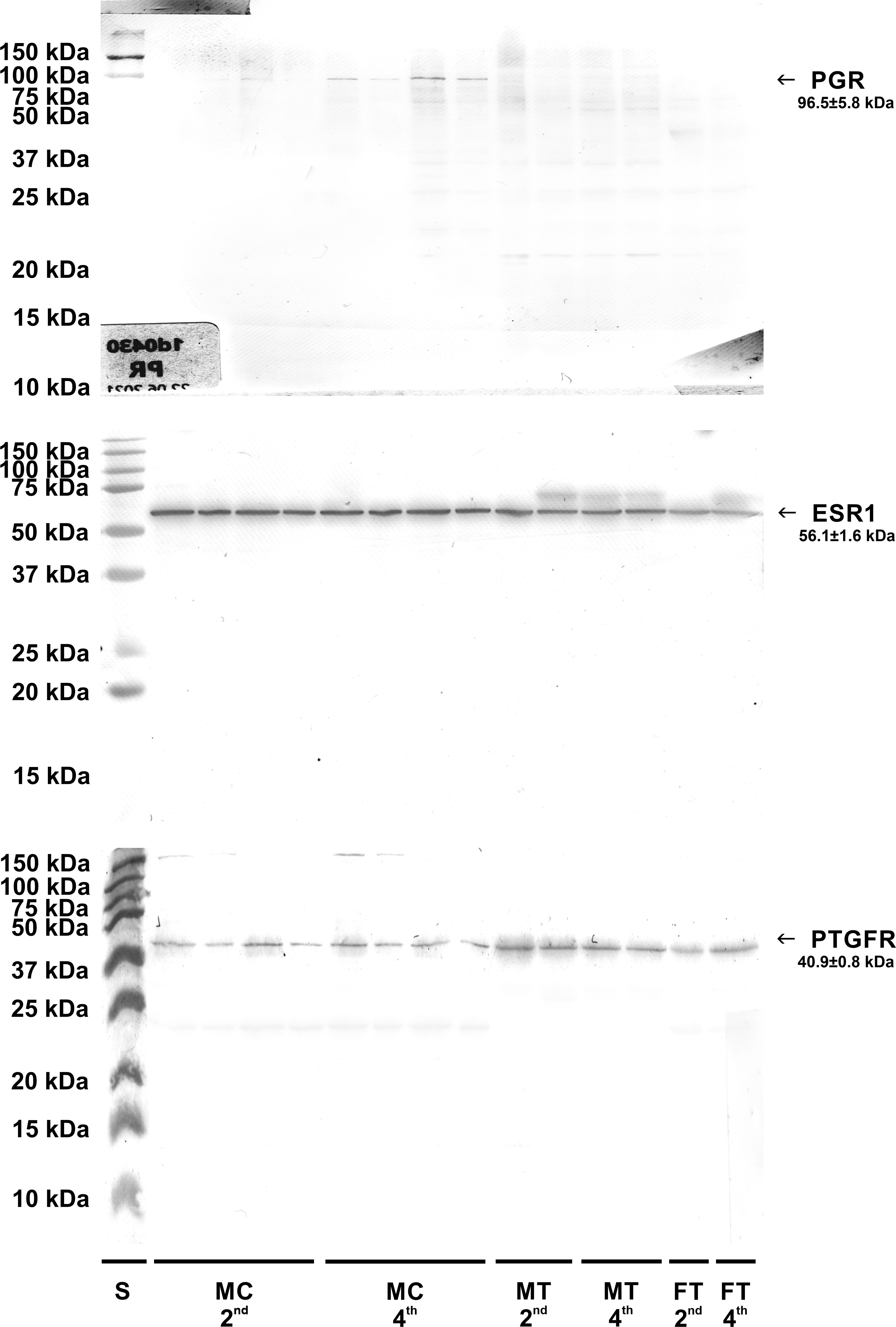

Supplement: Supplementary file 1 [file molecules-27-07420-s001.zip › Supplementary Figure S3_full-blot.jpg]
